# Cardiac resynchronization therapy: mechanisms of action and scope for further improvement in cardiac function

**DOI:** 10.1093/europace/euw136

**Published:** 2016-07-13

**Authors:** Siana Jones, Joost Lumens, S. M. Afzal Sohaib, Judith A. Finegold, Prapa Kanagaratnam, Mark Tanner, Edward Duncan, Philip Moore, Francisco Leyva, Mike Frenneaux, Mark Mason, Alun D. Hughes, Darrel P. Francis, Zachary I. Whinnett, the BRAVO Investigators, on behalf of the BRAVO Investigators, Nishi Chaturvedi, Wyn Davies, Boon Lim, David Lefroy, Nicholas S. Peters, Emma Coady, Katherine March, Suzanne Williams, Karikaran Manoharan, Nadia Do Couto Francisco, Vasco Miranda Carvalho, Andreas Kyriacou, Amelia Rudd, Nadiya Sivaswamy, Satnam Singh, Martin Thomas, Jon Swinburn, Paul Foley, Tim Betts, David Webster, Dominic Rogers, Tom Wong, Rakesh Sharma, Susan Ellery, Zaheer Yousef, Lisa Anderson, Mohamed Al-Obaidi, Nicky Margerison, Stephanie Barrett, Paul Kalra, Raj Khiani, Mark Dayer

**Affiliations:** 1 Institute of Cardiovascular Science, University College London, London WC1E 6BT, UK; 2 CARIM School for Cardiovascular Diseases, Maastricht University, Maastricht 6229 ER, The Netherlands; 3 NHLI—Cardiovascular Science, Imperial College London, National Heart and Lung Institute, The Hammersmith Hospital, B Block South, 2nd Floor, Du Cane Road, London W12 ONN, UK; 4 St Richards Hospital, Western Sussex Hospitals Foundation Trust, Chichester PO19 6SE, UK; 5 Bristol Royal Infirmary, Marlborough Street, Bristol BS2 8HW, UK; 6 Watford General Hospital, Vicarage Road, Watford WD18 0HB, UK; 7 Queen Elizabeth Hospital, Mindelsohn Way, Edgbaston, Birmingham B15 2WB, UK; 8 School of Medicine and Dentistry, University of Aberdeen, Aberdeen, UK; 9 Harefield Hospital, Hill End Road, Harefield, Middlesex UB9 6JH, UK

**Keywords:** Resynchronization, Cardiac resynchronization therapy, CRT, AV delay, CRT mechanisms

## Abstract

**Aims:**

Cardiac resynchronization therapy (CRT) may exert its beneficial haemodynamic effect by improving ventricular synchrony and improving atrioventricular (AV) timing. The aim of this study was to establish the relative importance of the mechanisms through which CRT improves cardiac function and explore the potential for additional improvements with improved ventricular resynchronization.

**Methods and Results:**

We performed simulations using the CircAdapt haemodynamic model and performed haemodynamic measurements while adjusting AV delay, at low and high heart rates, in 87 patients with CRT devices. We assessed QRS duration, presence of fusion, and haemodynamic response. The simulations suggest that intrinsic PR interval and the magnitude of reduction in ventricular activation determine the relative importance of the mechanisms of benefit. For example, if PR interval is 201 ms and LV activation time is reduced by 25 ms (typical for current CRT methods), then AV delay optimization is responsible for 69% of overall improvement. Reducing LV activation time by an additional 25 ms produced an additional 2.6 mmHg increase in blood pressure (30% of effect size observed with current CRT). In the clinical population, ventricular fusion significantly shortened QRS duration (Δ-27 ± 23 ms, *P* < 0.001) and improved systolic blood pressure (mean 2.5 mmHg increase). Ventricular fusion was present in 69% of patients, yet in 40% of patients with fusion, shortening AV delay (to a delay where fusion was not present) produced the optimal haemodynamic response.

**Conclusions:**

Improving LV preloading by shortening AV delay is an important mechanism through which cardiac function is improved with CRT. There is substantial scope for further improvement if methods for delivering more efficient ventricular resynchronization can be developed.

**Clinical Trial Registration:**

Our clinical data were obtained from a subpopulation of the British Randomised Controlled Trial of AV and VV Optimisation (BRAVO), which is a registered clinical trial with unique identifier: NCT01258829, https://clinicaltrials.gov

**What's new?**

- It has been assumed that ventricular resynchronization is the main mechanism through which cardiac resynchronization therapy improves cardiac function.
- We found that improving LV preloading by shortening AV delay plays a key role in the way CRT improves cardiac function. This information may be useful when designing methods to improve patient selection for CRT implantation.
- Current methods for delivering CRT do not fully reverse the impairment in ventricular activation, which occurs with left bundle branch block. The potential for extra improvement in cardiac function, with better ventricular resynchronization, has not previously been well characterized.
- We found that, encouragingly, there appears to be substantial scope for obtaining additional improvements in cardiac function if novel forms of CRT that provide more efficient ventricular resynchronization can be developed.

## Introduction

Cardiac resynchronization therapy (CRT) is an established treatment for patients with heart failure and left bundle branch block (LBBB) or complete heart block (CHB). However, despite treatment, morbidity and mortality remain high.^1^

The current assumption is that the predominant mechanism of action of CRT is ventricular resynchronization. However, improving LV filling (preload) by shortening effective atrioventricular (AV) delay to a more optimal value is also a potential mechanism through which CRT may improve function.^2–5^ Adjusting AV delay during CRT has previously been shown to have a powerful effect on acute haemodynamics.^3,6–8^ The relative importance of these two mechanisms has not been well characterized since differentiating the individual effect of each mechanism on measures of cardiac function is challenging.

Currently, routine delivery of CRT is via the coronary sinus in conjunction with endocardial right ventricular (RV) and right atrial leads. While CRT delivered in this way to patients with LBBB is proved beneficial, it does not appear to fully reverse the underlying conduction impairment.^9^ Therefore, an opportunity exists to improve the delivery of ventricular resynchronization therapy, and it remains unclear how much extra improvement in cardiac function could be expected with improved resynchronization.

In this study, we aimed to answer the following questions: First, what is the contribution of shortening AV delay to the overall improvement in cardiac function obtained with CRT? Second, what magnitude of additional improvement in cardiac function would be expected if methods for improving the delivery of ventricular resynchronization could be achieved?

We address these mechanistic questions regarding the physiology of the acute beneficial effects of CRT using both computer simulations and clinical data. The computer simulations allowed us to address questions which are difficult to answer using clinical data alone.

We then tested the hypotheses generated using the computer model in patients using high-precision haemodynamic measurements. This verification of the simulation data with clinical data is important.

## Methods

### Computer simulations

The CircAdapt computational model of the human heart and circulation^10,11^ was used to quantify the magnitude of improvement in cardiac function available through AV delay optimization only (i.e. without changing ventricular activation pattern) and in combination with different degrees of left ventricular (LV) resynchronization.

**Figure 1:**
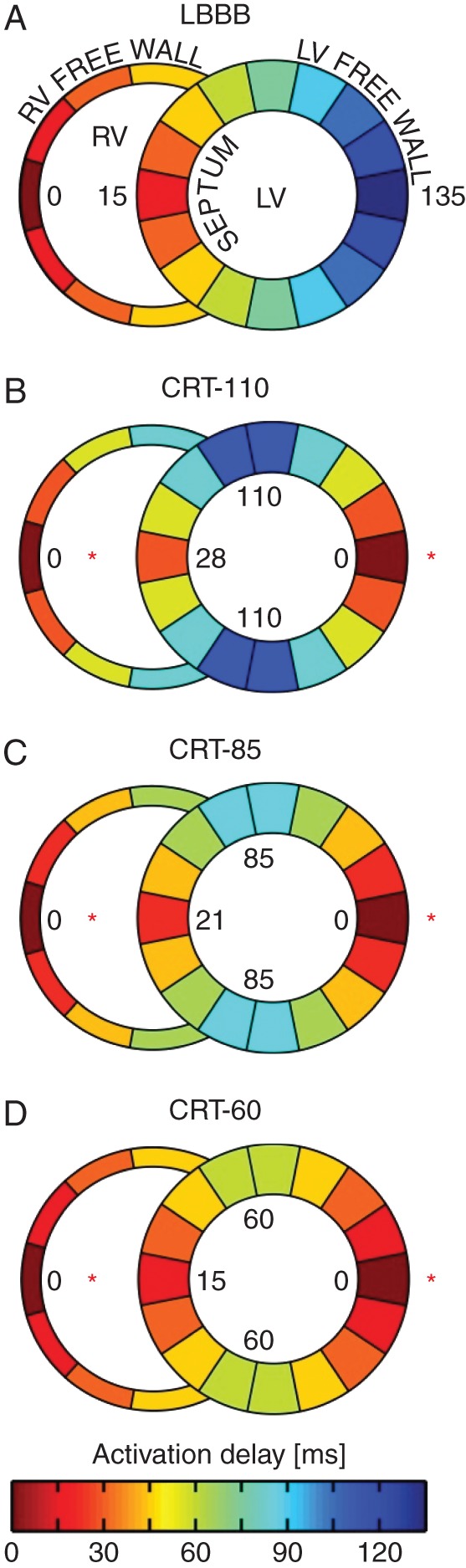
Simulated ventricular activation patterns. Four different LV activation states were simulated: (*A*) typical LBBB; (*B*) LV activation time of 110 ms (CRT-110), which represents the typical electrical resynchronization obtained with current CRT; and (*C* and *D*) additional 25 and 50 ms reduction in ventricular activation time, over and above that obtained with current CRT. This represents LV activation times of 85 ms (CRT-85) and 60 ms (CRT-60), respectively. Red stars indicate RV and LV lead positions.

First, a failing heart with LBBB was simulated (*Figure 1A*), intrinsic AV delay was prolonged to 220 ms, and heart rate was set to 80 bpm. Haemodynamic measurements for all other simulations were compared with this reference simulation. Next, we simulated the degree of ventricular resynchronization obtained with current methods for delivering CRT (*Figure 1B*), resulting in a 25 ms shortening of total LV activation time. Finally, two further simulations of more successful ventricular resynchronization were performed, i.e. additional 25 and 50 ms reductions in LV activation time (*Figure 1C* and *D*).

For all four LV activation patterns, we progressively shortened AV delay from 220 to 20 ms (decrements of 10 ms) and plotted LV stroke volume (SV) and aortic systolic blood pressure (SBP) as functions of AV delay.

The CircAdapt model describes the physiological interactions between its modules representing myocardial walls, cardiac valves, large blood vessels, peripheral resistances, and the pericardium.^10,12^ Simulations of mechanical and haemodynamic interventricular(VV) and AV interactions under normal and pathophysiological circumstances have been validated in previous studies.^10,13^ We describe this model further in the supplemental data file (Supplementary material online, *Appendix S1*).

### Study population

We recruited 87 consecutive patients with CRT devices, sinus rhythm, and LBBB or CHB, and a power calculation is provided online in Supplementary material online, *Appendix S2*. We adjusted AV delay at low and high heart rates.

This was a sub-study of the British Randomised Controlled Trial of AV and VV Optimisation (BRAVO, NCT01258829^14^). The BRAVO study was designed to compare echocardiography-guided optimization with haemodynamic optimization of AV and VV delays. All patients with a clinically indicated but previously implanted CRT device (EF < 35% at the time of implant) were included regardless of current LV function or the presence or absence of clinical response.^15^

All patients gave written informed consent for the study, which was approved by the local Research Ethics Committee.

For this sub-study, only patients with underlying LBBB QRS morphology or CHB (and therefore 100% RV pacing) were included. Left bundle branch block was defined as QRS duration above 120 ms and typical 12-lead electrocardiography morphology.^16^

#### Haemodynamic measurements

Blood pressure (BP) was measured using a Finometer device (Finapres Medical System, The Netherlands) yielding a continuous arterial pressure waveform.

In order to obtain a reliable assessment, we used the following protocol. We compared each tested AV delay to a reference AV delay of 120 ms and calculated the relative change in SBP. This was calculated as the difference between the mean of 8 beats immediately before and after the transition to the tested AV delay from the reference AV delay. We repeated this measurement a minimum of six times for each tested AV delay and used this to calculate the mean relative change in SBP for each tested delay.^2–4^ Tested AV delays were 40, 80, 140, 160, 200, 240, and 280 ms or up to an AV delay where ventricular pacing was withheld by the device. When possible, AAI mode pacing was also compared with the reference. The programmed AV delay yielding the greatest mean increase in BP was considered the optimum.

The protocol was carried out at two heart rates during atrial pacing: low heart rate (∼5 bpm above resting heart rate) and high heart rate (mean 103 ± 6 bpm). VV delay was kept constant throughout the measurements, and it was programmed to 0 ms or as close to this as the device would allow.

#### Assessment of conduction pattern and atrioventricular node decrementation

The ventricular activation pattern was assessed using the morphology of the surface electrocardiogram (ECG), which was collected at each programmed AV delay at each heart rate. We classified ventricular activation as follows:

- *Pure intrinsic conduction*: Ventricular activation results from ventricular activation only. This was assessed during AAI pacing mode.
- *CRT pacing with complete ventricular capture*: Ventricular activation occurs only as a result of the biventricular ventricular pacing stimuli. This was assessed while pacing with an AV delay of 40 ms.
- *Ventricular fusion*: Ventricular activation results from a combination of CRT pacing and intrinsic conduction. QRS morphology differs from that obtained during CRT pacing with complete ventricular capture and pure intrinsic conduction.

We calculated QRS duration using measurements from three separate QRS complexes obtained at each tested AV delay. Measurements were made using digitally stored electrograms with screen callipers at a paper speed of 100 ms. We measured from the rapid deflection of the QRS in order to avoid including the isoelectric period following the pacing spike in the QRS measurement.

AV node decrementation was considered to be present if ventricular fusion occurred at a longer programmed AV delay at high heart rate compared with low heart rate.

#### Patient categorization

We divided our study population according to pre-specified criteria based on the presence or absence of ventricular fusion and the conduction pattern offering the best haemodynamic response.

We subdivided patients who showed fused conduction according to whether the highest haemodynamic response occurred with or without fusion at low heart rate. We subdivided the group where the highest haemodynamic response occurred with fusion according to whether decrementation of intrinsic AV node conduction was present or absent. Therefore, the following four subgroups were obtained:

1. ‘No fusion’, CHB or very long intrinsic PR interval. Therefore, optimal AV delay occurred with biventricular pacing (BVP) at both rates. This group served as the control to assess the effect of increasing atrial pacing on optimal AV delay timing when ventricular fusion is not present.
2. ‘Optimum < fusion’, the AV delay with the highest haemodynamic response occurred at an AV delay without fusion at the low heart rate.
3. ‘Fused optimum, without AV node decrementation’, the highest haemodynamic response occurred with fusion at low heart rate, and increasing heart rate did not delay onset of fusion.
4. ‘Fused optimum with decrementation’, the highest haemodynamic response occurred with fusion at low heart rate and increasing heart rate delayed the onset of fusion (which occurred at a longer AV delay compared with the low heart rate).

### Statistical analysis

Data are presented as mean ± standard deviation (SD) or mean difference ± SD of difference. Data were examined for normality prior to further statistical analysis. Statistical comparisons of continuous data were made using repeated measures one-way analysis of variance (ANOVA) for within- or between-individual comparisons of more than two groups as appropriate. Between-group testing was performed if ANOVA was significant, paired *t*-tests for within-individual comparisons, and unpaired *t*-test for between-group comparisons. Comparisons of categorical data were made using a χ^2^ test.

## Results

### Computer simulations

#### Relative contribution of atrioventricular delay optimization and ventricular resynchronization

**Figure 2:**
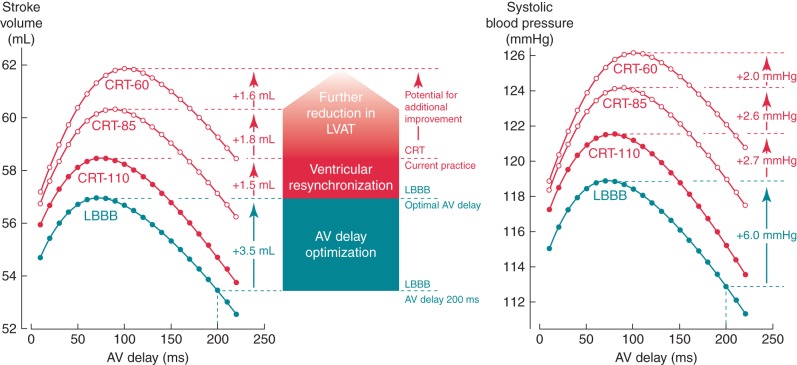
Simulation-based assessment of the relative importance of AV delay optimization and left ventricular activation time (LVAT) with current CRT methods and predicted impact of additional improvements in LV resynchronization. The left plot shows the relationship between AV delay and SV, and the right plot shows the relationship between AV delay and SBP. Four different ventricular activation states are displayed. Typical LBBB (LV activation time 135 ms). The typical electrical resynchronization obtained with current CRT [LV activation time of 110 ms (CRT-110)]. Additional 25 and 50 ms reductions in ventricular activation time, over and above that obtained with current CRT [LV activation times of 85 ms (CRT-85) and 60 ms (CRT-60)]. The Turquoise vertical arrow shows the improvement gained from optimizing AV timing relative to a baseline AV delay of 200 ms. Improvements gained from each additional reduction in LVAT are indicated by the red vertical arrows.

We adjusted AV delay in the CircAdapt model without changing the baseline LBBB-related dyssynchrony of ventricular activation. The effect on SV and BP depended on the starting AV delay (*Figure 2*). For example, using a PR interval of 201 ms (the average in our study population), AV delay optimization generated a 6 mmHg increase in SBP without changing the ventricular activation pattern.

During simulated CRT pacing (a 25 ms reduction in LV activation time compared with LBBB), we observed a further 2.7 mmHg increase in SBP on top of that obtained with AV delay optimization only. Therefore, for this example simulation with an intrinsic PR interval of 201 ms, AV delay optimization accounted for 69% of the overall available improvement in cardiac function (6/(6 + 2.7) × 100).

#### Potential for further improvements in cardiac function with improved delivery of ventricular resynchronization

A 25 ms reduction in LV activation time (*Figure 2*: CRT-85) relative to current standard CRT pacing (*Figure 2*: CRT-110) resulted in an additional 2.6 mmHg increase in SBP, while an additional 50 ms reduction in LV activation time (*Figure 2*: CRT-60) produced a 4.6 mmHg increase in SBP. With an intrinsic PR interval of 201 ms, these increases in SBP represent 30% ((2.6/8.7) × 100) and 53% ((4.6/8.7) × 100), respectively, of the total improvement observed with CRT using current methods for delivery and optimal AV delay.

### Clinical data

The characteristics of the 87 patients, recruited from 18 different centres within the UK, are shown in *Table 1*.

**Table 1:** Patient demographics

Demographic	% (*n* = 87)
Gender	
Male	78
Age (years ± SD)	67.3 ± 11.7
NYHA Functional Class	
II	93
III	7
Months post-implant ± SD	83 ± 143
Medication	
ACE/ARB inhibitors	87
Beta-Blockers	92
Digoxin	14
Furosemide	53
Spironolactone	41
Ischaemic aetiology	60
Intrinsic ECG measurements	(*n* = 69)
PR interval (ms)	203 ± 45
QRS duration (ms)	175 ± 25
Echocardiographic measurements	
LA dimension (cm)	4.5 ± 0.72
LVEDD (cm)	5.81 ± 1.14
LVESD (cm)	4.92 ± 1.10
Ejection fraction (%)	40.5 ± 10

Data are means ± SD or percentage of participants (%).

LA, left atrium; LVEDD, left ventricular end diastolic dimension; LVESD, left ventricular end systolic dimension.

#### Effect of heart rate on atrioventricular delay determined as optimal

**Figure 3:**
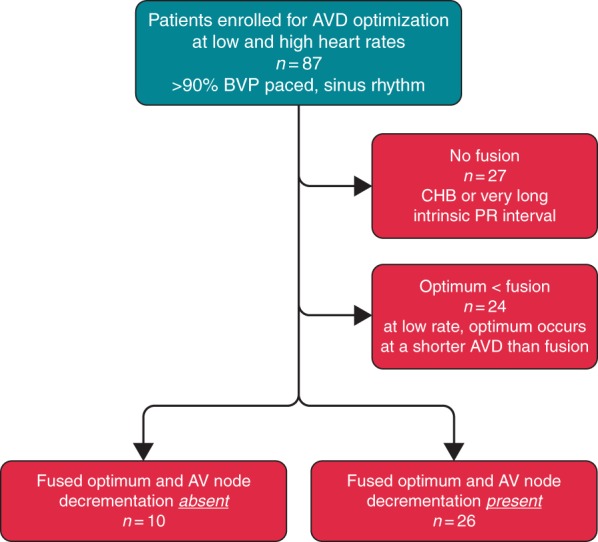
Categorization of patients. Patients were grouped according to their optimal AV delay determined by haemodynamic optimization at low and high heart rates and by the presence or absence of AV node decrementation. BVP and CHB.

Patients were categorized according to the pre-specified criteria described above (*Figure 3*). Mean difference between the AV delay optima determined at the low- and the high-paced heart rate was calculated for each group and compared between four groups (*P* = 0.09). *Post hoc* analysis revealed a significantly greater difference in Group 4 (Δ-30 ± 45 ms) vs. all other groups (Δ-9 ± 39 ms, *P* = 0.05). Diagrammatic representation of the difference between low and high heart rate optima is presented in the supplemental data (Supplementary material online, Figure S1).

The difference in AV delay determined as optimal between low and high heart rates is as follows for the four groups of patients.

Group 1. ‘No fusion’, comprising 27 patients with CHB (*n* = 18) or very long intrinsic PR interval (*n* = 9). No significant difference was found between the AV delay determined as optimal at the low and the high rates (Δ-9 ± 48 ms, *P* = 0.32).
Group 2. ‘Optimum < fusion’, comprising 24 patients in whom fusion only occurred at AV delays longer than the haemodynamic optimum. Despite the option to improve ventricular synchrony (with ventricular fusion), the AV delay producing the highest haemodynamic response occurred with BVP with complete ventricular capture, and there was no significant change in AV delay identified as optimal at the high heart rate (Δ-4 ± 28 ms, *P* = 0.46). This group comprises 40% of the population where fusion was available.
The AV delay at which fusion first occurred was significantly longer in Group 2, mean 231 ± 45 ms compared with Group 3 (mean 200 ± 33 ms) and Group 4 (203 ± 46 ms), *P* = 0.02.
Group 3. ‘Fused optimum, without AV node decrementation’, comprising 10 patients in whom the onset of fusion was not delayed by pacing at the higher heart rate. The optimal AV delay did not significantly differ between low and higher rate pacing (Δ-20 ± 34 ms, *P* = 0.096).
Group 4. ‘Fused optimum with decrementation’, comprising 26 patients in whom the low-rate optimum occurred during fusion and the higher heart rate delayed fusion by decrementing AV conduction. There was a significant shortening of the AV delay identified as optimal at the high heart rate (Δ-30 ± 45 ms, *P* = 0.002). Further analysis of this group showed that in the majority of patients (77%), the optimal AV delay switched from occurring in the fusion zone at low rate to CRT pacing with complete ventricular capture at the high rate, and this was significant by χ^2^ test (*P* = 0.009).

#### Ventricular resynchronization with current methods for delivering cardiac resynchronization therapy

**Figure 4:**
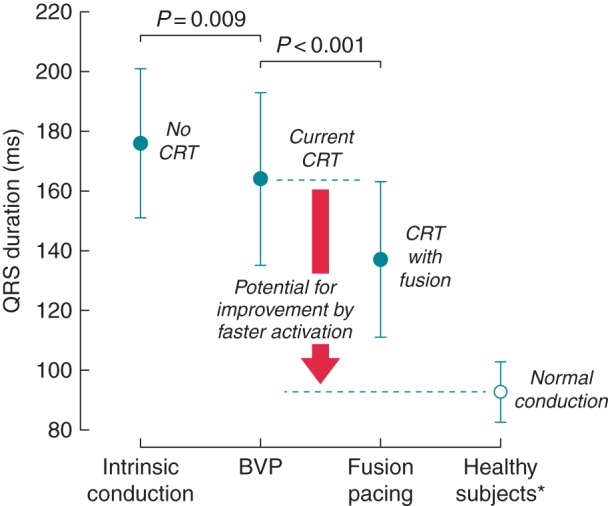
Untapped potential for ventricular resynchronization. QRS duration is shown during intrinsic conduction (no CRT), with CRT, and with CRT pacing allowing fusion of paced and intrinsic conduction, and previously published data for QRS duration from healthy subjects are shown for normal conduction.^17^

We quantified electrical ventricular resynchronization, in all patients who demonstrated fusion (regardless of group), by comparing QRS duration in three states (mean QRS ± SD): (a) intrinsic conduction (176 ± 25 ms), (b) CRT pacing with complete ventricular capture (164 ± 29 ms), and (c) BVP with fusion (137 ± 26 ms, *P* < 0.001). These values were compared with previously published mean QRS duration from healthy subjects^17^ (*Figure 4*). There was a significant difference in QRS duration between the three conduction states (three-way ANOVA, *P* < 0.001), and *post hoc* analysis showed that QRS duration was significantly narrower during fusion compared with CRT pacing with complete ventricular capture (Δ-27 ± 23 ms, *P* < 0.001) and significantly narrower with BVP than intrinsic conduction (Δ-12 ± 35 ms, *P* = 0.009). QRS duration and intrinsic PR interval are provided for each group in the supplemental data (see Supplementary material online, Table S1).

**Figure 5:**
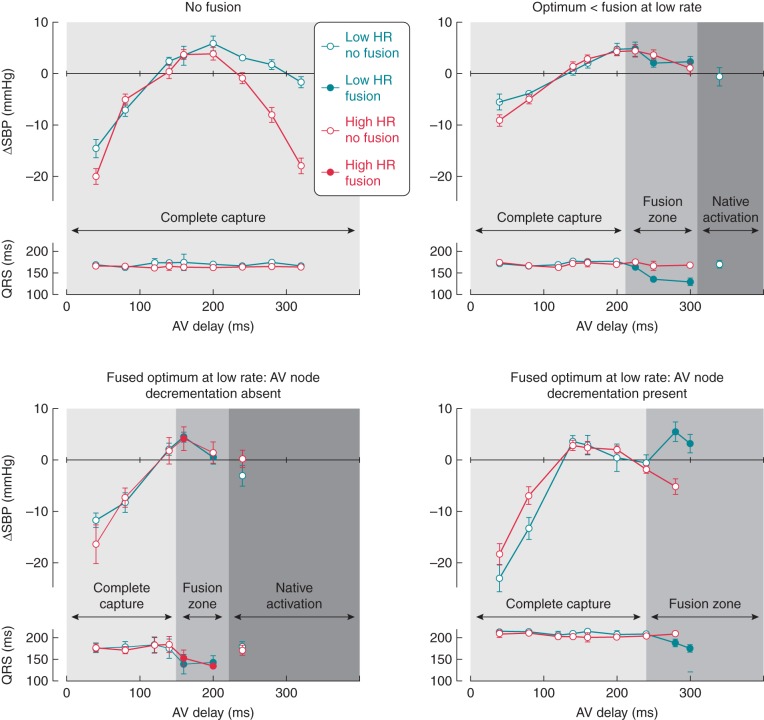
Example haemodynamic optimization data from an individual patient in each group. Low (Turquoise) and high (red) heart rates for each tested AV delay are shown. Each point represents the mean of 6–10 measurements, and the vertical bar represents the SD. Periods of complete capture, fusion, and native activation are shown, and AV delays that resulted in fusion are displayed as points filled with colour. QRS duration at each tested AV delay is plotted for low and high heart rates below the optimization curves. Abbreviations: heart rate (HR), change in SBP relative to value at AV delay 120 ms (ΔSBP).

Example haemodynamic data from each group are shown in *Figure 5*.

#### Assessment of haemodynamic impact of ventricular fusion

Group 4 allowed us to examine the association between fusion and acute haemodynamics since at low heart rate, the highest haemodynamic response occurred with fusion, but at high heart rate, the onset of fusion was delayed. Loss of fusion was associated with a mean decline in SBP, this is to say; mean relative SBP at the low heart rate optimal AV delay (fusion) minus the mean relative SBP at the same AV delay at high heart rate (without fusion) is Δ-2.5 mmHg, SDD 4.03, *P* = 0.004. We can say that, at a constant AV delay, fusion improved SBP by 2.5 mmHg and, in this group of patients, the haemodynamic improvement corresponded to a 22 ± 25 ms reduction in QRS duration.

## Discussion

Our findings from computer simulations and clinical data provide complementary evidence to suggest that, first, improving LV preloading by AV delay shortening is a fundamental mechanism through which CRT delivers its beneficial effect. Second, delivery of more efficient ventricular resynchronization has the potential to produce important additional improvements in cardiac function.

The simulations suggest that a 50 ms reduction in LV activation time would deliver an effect size equivalent to 53% of the total improvement observed with current methods for delivering CRT. The clinical data confirm that CRT currently does not produce complete electrical ventricular resynchronization and supports evidence from the simulations by showing that further reductions in QRS duration, produced by ventricular fusion, positively influence acute haemodynamic function.

### Improving atrioventricular timing is a critical component of the mechanism through which CRT delivers its beneficial effect

The results of the computational simulations suggest that AV delay optimization is fundamental to the mechanism through which CRT delivers its beneficial effect. The computational model of the human heart and circulation allowed us to independently quantify the effects of AV delay optimization and ventricular resynchronization. Both AV shortening and ventricular resynchronization had clear beneficial effects on acute haemodynamic function. The simulations demonstrated that the relative importance of the mechanisms of benefit is dependent on the baseline PR interval and the magnitude of the reduction in LV activation time with CRT. While these factors are patient specific, the results of the simulations suggest that improving LV preload through AV shortening is a key mechanism through which CRT currently improves cardiac function, when it is delivered to patients in sinus rhythm. Other factors such as interatrial conduction time may also affect the balance between the two mechanisms of benefit.

The findings from the clinical data support the findings from the computer simulations. In 40% of patients with ventricular fusion, and therefore with the potential to obtain improved ventricular resynchronization compared with complete ventricular capture, the highest haemodynamic response occurred at an AV delay with complete ventricular capture. If delivering improved ventricular electrical resynchronization was the sole mechanism through which CRT delivered its beneficial effect, then one would expect the optimal AV delay to always occur with fusion since this appears to deliver the most efficient ventricular activation. In this group of patients (Group 2), it appears the beneficial effects of fusion with respect to electrical ventricular resynchronization were outweighed by the negative impact on LV preload resulting from a suboptimal AV delay. In this group, fusion occurred at longer AV delays compared with the groups (3 and 4) where fusion resulted in the highest haemodynamic response (Supplementary material online, Table S1). Furthermore, in the group where there was a delayed onset of fusion with higher rate pacing (Group 4), the optimal AV delay shortened, rather than lengthened as would be expected if ventricular resynchronization was the only mechanism through which CRT delivers its beneficial effect.

The finding that shortening AV delay is an important mechanism through which CRT delivers its beneficial effect is supported by the findings of a sub-analysis of the COMPANION study, where patients with a longer PR interval were shown to obtain a greater relative risk reduction.^18^

Previous trials, reporting clinical outcomes, have suggested that AV optimization using electrical algorithms or echocardiographic methods, do not influence CRT outcome.^19^ In these studies, AV delay was shortened relative to sinus rhythm, in all patients including those in the control group where a nominal AV delay was programmed. Therefore, these studies were not designed to assess whether improving AV timing compared with sinus rhythm contributes to the overall mechanism through which CRT produces its beneficial effects.

### Potential for additional improvements in cardiac function with improved ventricular resynchronization

CRT pacing with complete ventricular capture produced only modest reductions in QRS duration (*Figure 4*) implying that CRT has only modest effects on ventricular electrical synchrony. Our findings are consistent with studies that have assessed the effect of CRT using ventricular activation mapping^20,21^ and large clinical trials reporting changes in QRS duration.^22^

The computer simulation data suggested that improvements in cardiac function could be obtained if methods for delivering more efficient ventricular resynchronization can be developed. Encouragingly, the findings from the simulations were supported by the clinical data. Ventricular fusion appeared to deliver more efficient ventricular activation; the QRS duration during fusion was significantly shorter. We observed a 2.5 mmHg improvement in BP in the presence of fusion compared with when fusion was not present, at the same AV delay in the same patient. The corresponding reduction in QRS duration with this BP improvement was 22 ms. This change in SBP was similar to the magnitude of change observed with the simulations (2.7 mmHg for a 25 ms reduction in LV activation time). Our finding of a reduction in QRS duration and improvement in acute haemodynamic function during fusion is consistent with the findings of other investigators.^23^

However, even with fusion, ventricular electrical activation appears to be far from normal, with mean QRS duration considerably longer than reports from healthy subjects (93 ± 10 ms)^17^ (*Figure 4*) suggesting scope for even greater improvements in cardiac function.

### Clinical application of these findings

These findings are relevant to clinical practice for the following reasons. Firstly, understanding the true ratio of the mechanisms of CRT benefit may allow better characterization of the patient population most likely to obtain benefit. Given that improving AV timing appears to be the dominant mechanism of benefit under most circumstances, better characterizing AV mistiming in addition to electrical measures of ventricular dyssynchrony may improve patient selection. Secondly, the finding that there is considerable scope for delivering improved ventricular resynchronization should stimulate the scientific community to develop methods for improving the way therapy is delivered.

#### Suitability of systolic blood pressure as marker of acute cardiac function

We chose SBP as our marker of acute cardiac function for the following reasons:

- It is an extra cardiac measure and therefore represents the net effect on cardiac function occurring as a result of the changes in pacemaker settings.
- Multiple repeated measurements can be made in order to minimize the effect of noise, which is present in all measures of cardiac function. This allows reproducible results to be obtained.^24^
- Changes in BP reflect changes in other markers of cardiac function such as LV dp/dt_max_ and aortic flow.^25^

In patients with heart failure, it is known that higher BP is associated with better outcomes.^26^ Cardiac resynchronization therapy when delivered to patients with LBBB is associated with acute improvements in SBP.^7^ Sustained improvements in SBP were observed in the randomized studies assessing longer-term outcomes with CRT. For example in the Care-HF study, a 6.3 mmHg increase in SBP was observed in the treatment arm 18 months follow-up.^1^

### Limitations

This study did not assess clinical endpoints; it was designed to make high-resolution comparisons between AV delay settings at two different paced heart rates in a manner that explored physiological mechanisms. Testing for an effect on clinical endpoints would require a large randomized trial. Such a trial would be justified once candidate methods of delivering better ventricular resynchronization have been tested in studies that could be acute but must be bias resistant.^27^

We used the well-established CircAdapt^10,11^ computational system to address mechanistic questions that could not be directly addressed in humans. All such computations should be interpreted with caution. However, for the questions which could be answered clinically as well as computationally, the results from both approaches were concordant.

The patients recruited into the clinical part of the study all had their CRT devices implanted prior to inclusion in the study. It is likely that they will have already experienced remodelling following device implantation; it is possible that this could have affected the results of the study.

It is possible that LV lead position could influence the optimal AV delay, and we did not aim to address this question in the present study. Lead positions were kept constant in individual patients throughout the study.

The AV delay providing the highest haemodynamic response is patient specific. It is likely that this is dependent on many factors which we did not aim to investigate in the present study. In order to keep these factors constant, we calculated relative change in optimal AV delay in individual patients, thereby keeping lead position and cardiac substrate constant when investigating the effect of changing heart rate and the presence or absence of fusion.

## Conclusions

Current methods for delivering CRT result in modest reductions in QRS duration, suggesting incomplete reversal of electrical dyssynchrony. With current methods for delivering CRT, improving LV preloading by AV delay shortening appears to be an important mechanism through which therapy improves cardiac function. Our computational calculations and clinical data suggest substantial further scope for delivering improvements in cardiac function if novel forms of CRT could be developed to provide more efficient ventricular resynchronization.

## Supplementary material

Supplementary material is available at *Europace* online.

## Funding

This work was supported by the UK's cardiovascular charity, the British Heart Foundation (BHF) (SP/10/002/28189) and the National Institute for Health Research. Z.W. (FS/13/44/30291), D.F. (FS/10/038), and A.S. (FS/11/92/29122) receive funding from the BHF. J.L. receives funding from the Dr E. Dekker program of the Dutch Heart Foundation (2012T010). Funding to pay the Open Access publication charges for this article was provided by British Heart Foundation.

**Conflict of interest:** D.P.F. and Z.I.W. are named as inventors on a patent filed by Imperial College London on methods for haemodynamic optimization of CRT pacemakers that reduce uncertainty.

## Supplementary Material

Supplementary Data
