# Supplementary material for: Cardiac resynchronization therapy: mechanisms of action and scope for further improvement in cardiac function
Source: Europace. 2016 Jul 13;19(7):1178–86. doi: 10.1093/europace/euw136 (PMC5834145; doi:10.1093/europace/euw136)
Supplement: Supplementary Data [file euw136_supp.docx]

# Supplemental Information

## Appendix 1

### Detailed description of the CircAdapt computer model used to generate simulation data

The CircAdapt model describes the physical and physiological interactions between its modules representing myocardial walls, cardiac valves, large blood vessels, peripheral resistances, and the pericardium.[^1^](#_ENREF_1)^,^ [^2^](#_ENREF_2) It enables realistic and real-time simulation of beat-to-beat cardiovascular mechanics and hemodynamics under a wide variety of physiological and pathophysiological conditions. Simulations of mechanical and hemodynamic interventricular and atrioventricular interactions under normal and pathophysiological circumstances have been validated in previous studies.[^2^](#_ENREF_2)^,^ [^3^](#_ENREF_3) Furthermore, CircAdapt enables realistic simulation of regional myocardial deformation in failing hearts with heterogeneous electromechanical tissue properties in the ventricular walls and during CRT.[^4-6^](#_ENREF_4) The model’s source code is freely accessible through an open access journal[^6^](#_ENREF_6) or [www.circadapt.org](http://www.circadapt.org).

First a failing heart with LBBB was simulated as described previously.[^4^](#_ENREF_4) In brief, several model parameters were changed to match average patient data that were measured in a typical cohort of CRT candidates; ventricular contractility was decreased to 40% of its normal value so that LV ejection fraction was 23%. A typical LBBB-like pattern of ventricular activation was imposed (Figure 1 panel A); and intrinsic AV delay was prolonged to 220ms. Heart rate was set to 80 bpm and systemic peripheral resistance and total circulating blood volume were adjusted so that mean arterial pressure and cardiac output were 92 mmHg and 4.2 L/min, respectively. The resulting reference simulation was used as starting point for all AV optimization and resynchronization simulations.

## Appendix 2

**Power Calculation**

We have previously shown that the reproducibility of AV delay optimization performed at two different visits using a protocol of calculating the relative change in SBP compared to a reference AV delay of 120ms and performing repeated alternations at each tested AV delay (six alternations) is -2.5ms and the standard deviation of the difference is ±12.4ms.[^7^](#_ENREF_7) Therefore, to have 90% power at the 5% significance level, to be able to detect a difference in AV delay optimum of 20ms between repeated optimization requires 5 patients.

We deliberately recruited more patients to ensure that each sub-group would be adequately represented and include at least 5 patients.

## Supplemental Table

| Group (n) |  |  |  | QRS duration (mean ±SD) | | | |
| --- | --- | --- | --- | --- | --- | --- | --- |
|  | Intrinsic PR interval (ms) | AVD fusion  (ms) | Intrinsic conduction (ms) | CRT with complete capture (ms) | Ventricular fusion (ms) | ΔQRS  QRS during CRT with complete capture-QRS during fusion (ms) |  |
| 1 (27) | NA | NA | NA | 174 ±33 | NA | NA |  |
| 2 (24) | 203 ±41 | *231±45 | 179 ±18 | 169 ±32 | 138 ±27 | -31±23 |  |
| 3 (10) | 174 ±19 | 200±33 | 171 ±30 | 147 ±28 | 126 ±28 | -22±22 |  |
| 4 (26) | 197 ±41 | 203±46 | 175 ±28 | 166 ±25 | 141 ±24 | -25±23 |  |
| All patients (87) | 203 ±45 | 213±45 | 175 ±25 | 167 ±30 | 137 ±26 | -27±23 |  |

**Supplemental Table 1.**

***The AV delay at which fusion was first observed was, significantly longer in group 2 compared to group 3 and 4 together (Δ31ms, p=0.02).**

**Mean PR interval, AVD first allowing ventricular fusion and QRS duration.**

Mean PR intervals during sinus rhythm with intrinsic conduction are shown for each of the four groups. Mean AV delay (AVD) which was the shortest allowing fusion of ventricular conduction is given for each group. Mean QRS durations recorded during atrial pacing at the low heart rate are shown for each of the following states: Intrinsic conduction (AAI pacing), CRT pacing with complete ventricular capture and fused ventricular activation (CRT pacing and intrinsic conduction via His Purkinjee system).

## Supplemental Figure


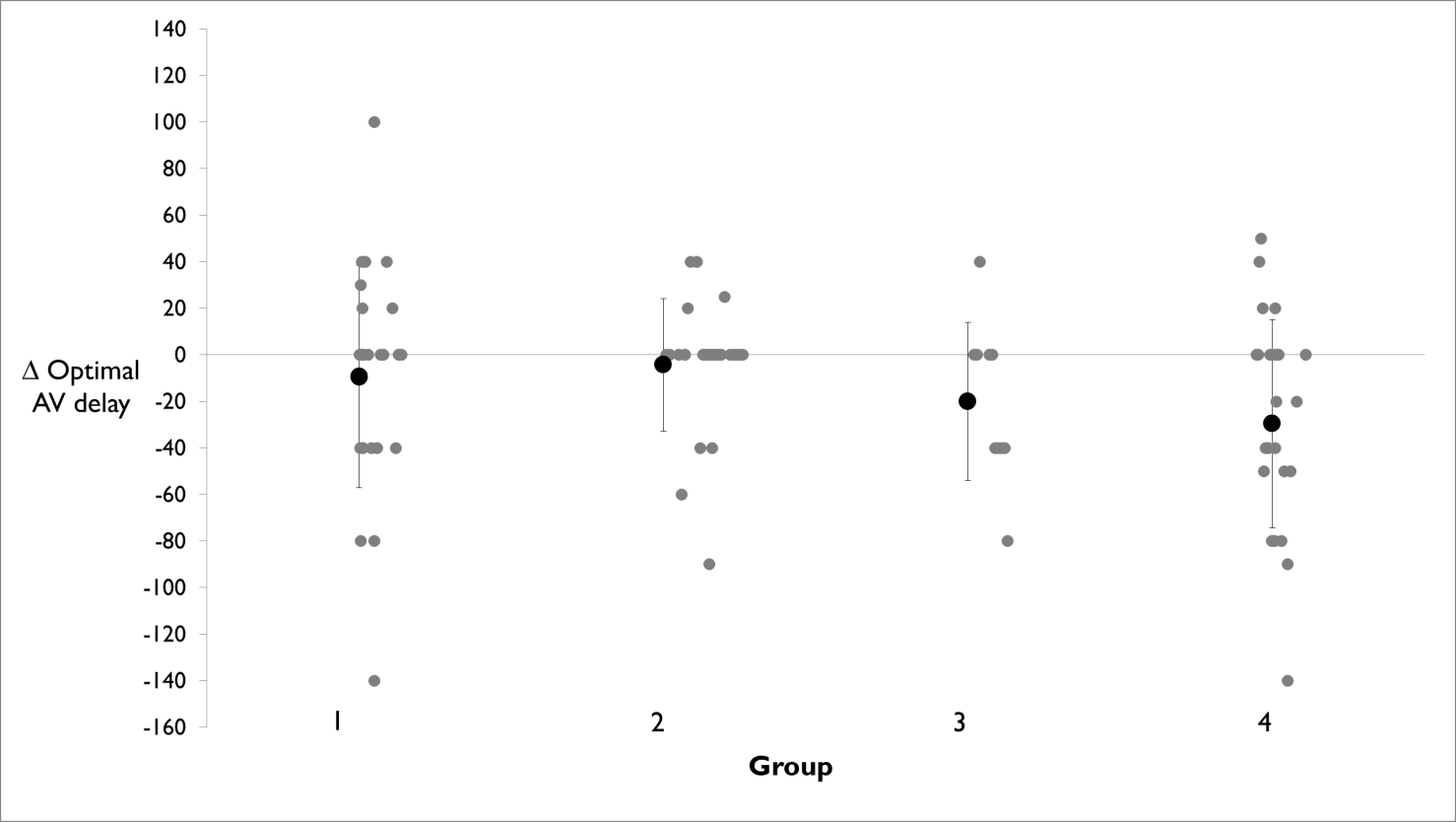
**Supplemental Figure 1**

**Difference in AVD determined as optimal with change in heart rate**

The Δ optimal AV delay (optimal AVD at high heart rate – Optimal AVD at low heart rate) is shown for each patient. The data is displayed according to the four groups into which patients were categorized : Group 1: No fusion, Group 2: optimum < fusion, Group 3: fused optimum no decrementation, Group 4: fused optimum with decrementation. Individual differences are represented by grey dots and the average for each group is represented as a large black dot. Vertical bars represent standard deviation of the whole groups’ deltas.

**References**

[1] Arts T, Delhaas T, Bovendeerd P, Verbeek X, Prinzen FW. Adaptation to mechanical load determines shape and properties of heart and circulation: the CircAdapt model. *American journal of physiology Heart and circulatory physiology* 2005; **288**: H1943-54.

[2] Lumens J, Delhaas T, Kirn B, Arts T. Three-wall segment (TriSeg) model describing mechanics and hemodynamics of ventricular interaction. *Ann Biomed Eng* 2009; **37**: 2234-55.

[3] Lumens J, Blanchard DG, Arts T, Mahmud E, Delhaas T. Left ventricular underfilling and not septal bulging dominates abnormal left ventricular filling hemodynamics in chronic thromboembolic pulmonary hypertension. *American journal of physiology Heart and circulatory physiology* 2010; **299**: H1083-91.

[4] Lumens J, Ploux S, Strik M, Gorcsan J, 3rd, Cochet H, Derval N, et al. Comparative electromechanical and hemodynamic effects of left ventricular and biventricular pacing in dyssynchronous heart failure: electrical resynchronization versus left-right ventricular interaction. *Journal of the American College of Cardiology* 2013; **62**: 2395-403.

[5] Lumens J, Tayal B, Walmsley J, Delgado-Montero A, Huntjens PR, Schwartzman D, et al. Differentiating Electromechanical From Non-Electrical Substrates of Mechanical Discoordination to Identify Responders to Cardiac Resynchronization Therapy. *Circ Cardiovasc Imaging* 2015; **8**: e003744.

[6] Walmsley J, Arts T, Derval N, Bordachar P, Cochet H, Ploux S, et al. Fast Simulation of Mechanical Heterogeneity in the Electrically Asynchronous Heart Using the MultiPatch Module. *PLoS computational biology* 2015; **11**: e1004284.

[7] Whinnett ZI, Davies JE, Nott G, Willson K, Manisty CH, Peters NS, et al. Efficiency, reproducibility and agreement of five different hemodynamic measures for optimization of cardiac resynchronization therapy. *International journal of cardiology* 2008; **129**: 216-26.
